# COVID-19 hospitalizations in five California hospitals: a retrospective cohort study

**DOI:** 10.1186/s12879-021-06640-4

**Published:** 2021-09-10

**Authors:** Miriam Nuño, Yury García, Ganesh Rajasekar, Diego Pinheiro, Alec J. Schmidt

**Affiliations:** 1 Department of Public Health Sciences, University of California, One Shields Avenue, Medical Sciences 1C, Davis, CA 95616 USA; 2 Department of Surgery, University of California, Davis, USA; 3 UNICAP, ICAM-Tech International School, Universidade Católica de Pernambuco, Recife, Brazil; 4 Centro de Investigación en Matemática Pura y Aplicada (CIMPA), University of Costa Rica, San José, Costa Rica

**Keywords:** Severe acute respiratory syndrome coronavirus 2, Coronavirus disease 2019, Hospitalization, Comorbidities, Intensive care unit, In-hospital mortality, Composite outcome

## Abstract

**Background:**

The novel coronavirus pandemic has had a differential impact on communities of color across the US. The University of California hospital system serves a large population of people who are often underrepresented elsewhere. Data from hospital stays can provide much-needed localized information on risk factors for severe cases and/or death.

**Methods:**

Patient-level retrospective case series of laboratory-confirmed COVID-19 hospital admissions at five UC hospitals (N = 4730). Odds ratios of ICU admission, death, and a composite of both outcomes were calculated with univariate and multivariate logistic regression based on patient characteristics, including sex, race/ethnicity, and select comorbidities. Associations between comorbidities were quantified and visualized with a correlation network.

**Results:**

Overall mortality rate was 7.0% (329/4,730). ICU mortality rate was 18.8% (225/1,194). The rate of the composite outcome (ICU admission and/or death) was 27.4% (1298/4730). Comorbidity-controlled odds of a composite outcome were increased for age 75–84 (OR 1.47, 95% CI 1.11–1.93) and 85–59 (OR 1.39, 95% CI 1.04–1.87) compared to 18–34 year-olds, males (OR 1.39, 95% CI 1.21–1.59) vs. females, and patients identifying as Hispanic/Latino (OR 1.35, 95% CI 1.14–1.61) or Asian (OR 1.43, 95% CI 1.23–1.82) compared to White. Patients with 5 or more comorbidities were exceedingly likely to experience a composite outcome (OR 2.74, 95% CI 2.32–3.25).

**Conclusions:**

Males, older patients, those with multiple pre-existing comorbidities, and those identifying as Hispanic/Latino or Asian experienced an increased risk of ICU admission and/or death. These results are consistent with reported risks among the Hispanic/Latino population elsewhere in the United States, and confirm multiple concerns about heightened risk among the Asian population in California.

**Supplementary Information:**

The online version contains supplementary material available at 10.1186/s12879-021-06640-4.

## Background

Severe acute respiratory syndrome coronavirus 2 (SARS-CoV-2), the virus causing coronavirus disease 2019 (COVID-19) has been a devastating pandemic, with over 91 million confirmed cases and nearly 2 million deaths worldwide [1]. In the United States, the state of California (hereafter referred to as CA) leads the charts for the worse surge across the nation, with a total of 70,561 confirmed cases per million residents, 1176 confirmed deaths per million residents, and potentially 1173 ICU beds available for a population of nearly 40 million (January 12, 2021).

Advanced age, chronic underlying conditions (including hypertension, diabetes, chronic kidney disease, among others), and being male are associated with an increased risk of hospitalization and death from COVID-19 [2–14]. There is additional, mounting evidence that some racial and ethnic minority groups are at disproportionate risk [3, 5, 6, 11]. Nation-wide, the Black/African American, Hispanic/Latino, Asian, and American Indian/Alaskan Native (AIAN) populations have seen elevated crude rates of infection, hospitalization, and/or mortality compared to the White population [15]. In trends similar to other states, estimates from CA indicate that the Hispanic/Latino population make up 55% of confirmed cases and 47% of deaths and only 39% of the total population the Black/African American population represent 4% of confirmed cases and 7% of deaths and only 6% of the total population; and the Asian population represent 7% of all cases but 12% of all deaths. Multiple informal analyses report that Asians have low rates 7of confirmed cases but concerningly high case fatality rates [16], but few studies have been large enough to capture associated risk factors in this population.

More diverse, localized information is needed about who is being hospitalized with COVID-19 and their outcomes in the United States, and California in particular, especially since the second surge in the last half of 2020. This study describes the demographic characteristics, baseline comorbidities, and outcomes of patients hospitalized with COVID-19 in five acute care medical centers in the University of California Health System.

## Methods

### Data collection and cohort identification

In this retrospective cohort study, we identified patients who were hospitalized with laboratory-confirmed SARS-CoV-2 infections at any of the five UC Health hospitals between December 13, 2019 and January 6, 2021 by extracting electronic medical records (EMRs) from The COVID Research Data Set (CORDS). The index date corresponds to the first confirmed COVID-19 diagnosis. A hospitalization was included if test confirmation occurred within 21 days or during an inpatient admission. SARS-CoV-2 positive status was determined by PCR or SNOMED code 840539006 (Disease caused by Severe acute respiratory syndrome coronavirus 2) during their earliest hospitalization (Additional file 1: Table S1).

Patient characteristics, including race/ethnicity, sex, comorbidities, and clinical outcomes were collected. Data was queried on January 8, 2021 and tabulated by January 10, 2021. We captured comorbidities using the *International Statistical Classification of Diseases and Related Health Problems, Tenth Revision* (*ICD*-*10*) classification (Additional file 1: Table S2). Self-identified race/ethnicity was categorized into White, Hispanic/Latino, Black/African American, and Asian; Other includes AIAN (n = 9, 0.19%), Native Hawaiian or Other Pacific Islander (n = 53, 1.12%), Other (n = 169, 3.57%), Unknown (n = 246, 5.20%), and those that identified with multiple race/ethnicity (n = 46, 0.97%).

Comorbidities included cancer, cardiac disease, cerebrovascular disease, coagulopathy, deficiency anemia, depression, diabetes, drug use disorders, HIV/Aids, hypertension, hypothyroidism, liver disease, neurological conditions, obesity, paralysis, pregnancy, psychoses, pulmonary disease, renal failure, collagen vascular disease, smoking status, and solid organ transplantation. Comorbidities documented 2 months prior to COVID-19 confirmation, during hospitalization, or 2 months post discharge were considered. The 2 month post-discharge observation period to capture comorbidities was deemed reasonable given lags in data entry due to billing. A comorbidity score represented the sum of any of these conditions. The lowest score of a 0 (n = 1806, 38.2%) was assigned to patients without documented comorbidities. The co-occurrence of comorbidities was illustrated through correlation networks. The UC Davis implementation of the University of California COVID Research Data Set (CORDS) was determined exempt from human subject protection under IRB protocol 1604619–1. Informed Consent was not deemed necessary for de-identified data by Nicholas and Kent Anderson at the UC Davis Clinical and Translational Science Award (CTSC) Biomedical Informatics Program.

### Statistical analysis

Univariate logistic regression was used to calculate the crude odds ratio (OR) of ICU admission and mortality events to discharge from the hospital. Multivariable logistic regression was used to estimate the odd ratio of the composite outcome of ICU admission and/or death during hospitalization to discharge from the hospital. The multivariable model adjusted for age in years at admission, sex, and race/ethnicity. Odds ratios and 95% confidence intervals (95% CI) were reported.

We measured the strength of comorbidity associations with the Pearson’s correlation coefficient $(\Phi$) for binary variables. The correlation coefficient between a pair of comorbidities, $\Phi ij$_,_ was calculated according to Equation (1) below.

1 $$\Phi ij = \frac{CijN - PiPj}{{\sqrt {PiPj\left( {N - Pi} \right)\left( {N - Pj} \right)} }}$$

where $Cij$ corresponds to the number of patients affected by both comorbidities, $N$ is the total number of patients in the study, and $Pi$ is the prevalence of the *i*th comorbidity. The distribution of $\Phi$ values represent all disease pairs where $Cij$ > 0. The correlation for the dichotomous variable is denoted by $\Phi$ and a t-test is used to determine the significance of $\Phi$ ≠ 0 [17].

We visualized comorbidities with correlation ($\Phi$ > 0) larger than expected by chance for the entire cohort, as well as stratified by composite outcome.

We described patterns in cumulative hospitalizations during the study period over time by using change-point analysis to discern specific time points where abrupt changes in the mean occurred. This algorithm detects multiple points of change in time-ordered data through binary segmentation [18–20]. First, we applied a change-point statistical test to the entire time series of hospitalization cases. Then, we split the series into two new ones when a point change was found. We repeated this procedure several times until there were no further significant change points. Statistical analyses were conducted using SAS software, version 9.4 (SAS Institute, Cary, NC, USA), the *changepoint* package (binary segmentation algorithm) in R version 4.0.1 (R Core Team 2020), and Python. Data were analyzed from January 8 to January 20, 2021.

## Results

A total of 4,730 hospitalized patients with positive SARS-CoV-2 assays were included (median age 61 years, IQR: 46–73) (Table 1). 56.4% were male; one patient was missing sex data. A total of 339 (7.2%) identified as Black/African American, 2161 (45.7%) as Hispanic/Latino, 489 (10.3%) as Asian, 523 (11.1%) were documented as Other/Unknown, and 1218 (25.8%) identified as White. A moderate fraction of patients did not have documented comorbidities (n = 1806, 38.2%). The most common comorbidities were hypertension (35.2%), cardiac disease (33.3%), diabetes (24.0%), pulmonary disease (17.8%), obesity (17.3%), and renal failure (15.3%). Coagulopathy (11.8%), smoking (10.2%), depression (9.4%), liver disease (8.8%), and cerebrovascular disease (7.8%) were also reported. Among the possible 22 comorbidities documented for each individual patient, we found that 457 (9.7%) patients had one, 488 (10.2%) had two, 468 (9.9%) had three, 430 (9.1%) had four, 355 (7.5%) had five, and 726 (15.4%) had six or more comorbidities.

**Table 1:** Characteristics and comorbidities of 4730 patients with COVID-19 hospitalized in UC Health Hospitals

Characteristic	No. patients n = 4730	ICU admission		In-hospital mortality	
		Cases (%) n = 1194 (25.2)	Crude OR (95% CI)	Cases (%) n = 329 (7.0)	Crude OR (95% CI)
Age in years, n (%)					
18–34	587 (12.4)	124 (21.1)	Reference	10 (1.7)	Reference
35–54	1204 (25.5)	287 (23.8)	1.17 (0.92–1.49)	43 (3.6)	2.14 (1.11–4.53)
55–74	1861 (39.3)	514 (23.8)	1.43 (1.14–1.79)	125 (6.7)	4.15 (2.28–8.50)
75–84	581 (12.3)	160 (27.6)	1.42 (1.09–1.86)	76 (13.1)	8.68 (4.66–18.04)
85+	497 (10.5)	109 (21.9)	1.05 (0.78–1.40)	75 (15.1)	10.25 (5.49–21.34)
Sex, n (%)^g^					
Female	2061 (43.6)	452 (21.9)	Reference	120 (5.8)	Reference
Male	2668 (56.4)	742 (27.8)	1.37 (1.20–1.57)	209 (7.8)	1.38 (1.09–1.74)
Race/ethnicity, n (%)					
White	1218 (25.6)	284 (23.3)	Reference	77 (6.3)	Reference
Black/African American	339 (7.2)	83 (24.5)	1.06 (0.80–1.40)	30 (8.9)	1.44 (0.91–2.21)
Hispanic/Latino	2161 (45.7)	551 (25.5)	1.13 (0.96–1.33)	144 (6.7)	1.06 (0.80–1.41)
Asian	489 (10.3)	123 (25.2)	1.11 (0.86–1.41)	46 (9.4)	1.54 (1.05–2.24)
Other/Unknown	523 (11.1)	153 (29.3)	1.36 (1.08–1.71)	32 (6.1)	0.97 (0.62–1.46)
Comorbidities, n (%)					
No comorbidity	1806 (38.2)	335 (18.6)	0.55 (0.47–0.63)	82 (4.5)	0.52 (0.40–0.66)
Cancer	191 (4.0)	45 (23.6)	0.91 (0.64–1.27)	20 (10.5)	1.60 (0.97–2.52)
Cardiac disease	1573 (33.3)	582 (37.0)	2.44 (2.13–2.80)	188 (12.0)	2.90 (2.32–3.65)
Cerebrovascular disease	367 (7.8)	160 (43.6)	2.49 (2.00–3.09)	63 (17.2)	3.19 (2.35–4.28)
Coagulopathy	560 (11.8)	246 (42.9)	2.66 (2.22–3.19)	91 (16.3)	3.21 (2.46–4.14)
Deficiency anemia	264 (5.6)	80 (30.3)	1.31 (0.99–1.71)	24 (9.1)	1.37 (0.86–2.07)
Depression	444 (9.4)	116 (26.1)	1.05 (0.84–1.31)	31 (7.0)	1.01 (0.67–1.45)
Diabetes	1137 (24.0)	399 (35.1)	1.90 (1.65–2.20)	120 (10.6)	1.91 (1.51–2.41)
Drug use disorder	242 (5.1)	65 (26.9)	1.09 (0.81–1.46)	12 (5.0)	0.69 (0.36–1.19)
HIV/Aids	444 (9.4)	6 (15.4)	0.54 (0.20–1.19)	3 (7.7)	1.12 (0.27–3.11)
Hypertension	1664 (35.2)	527 (31.7)	1.67 (1.46–1.91)	178 (10.7)	2.31 (1.85–2.90)
Hypothyroidism	340 (7.2)	100 (29.4)	1.26 (0.98–1.60)	35 (10.3)	1.60 (1.09–2.82)
Liver disease	416 (8.8)	149 (35.8)	1.75 (1.41–2.16)	50 (12.0)	1.98 (1.42–2.70)
Neurological conditions	709 (15.0)	314 (44.3)	2.84 (2.40–3.35)	130 (18.3)	4.31 (3.40–5.46)
Obesity	816 (17.3)	275 (33.7)	1.66 (1.41–1.95)	56 (6.9)	0.98 (0.72–1.31)
Paralysis	100 (2.1)	50 (50.0)	3.05 (2.05–4.54)	16 (16.0)	2.63 (1.47–4.42)
Pregnancy	171 (3.6)	21 (12.3)	0.40 (0.25–0.63)	3 (1.8)	0.23 (0.06–0.62)
Psychoses	108 (2.3)	20 (18.5)	0.67 (0.40–1.07)	3 (2.8)	0.38 (0.09–1.01)
Pulmonary disease	844 (17.8)	330 (39.1)	2.25 (1.92–2.63)	122 (14.5)	3.00 (2.36–3.80)
Renal failure	723 (15.3)	255 (35.3)	1.78 (1.50–2.11)	96 (13.3)	2.48 (1.92–3.18)
Collagen vascular disease*	150 (3.2)	45 (30.0)	1.28 (0.89–1.81)	12 (8.0)	1.17 (0.61–2.05)
Smoker	480 (10.2)	147 (30.6)	1.35 (1.10–1.66)	53 (11.0)	1.79 (1.30–2.42)
Solid organ transplantation	221 (4.7)	72 (32.6)	1.46 (1.09–1.94)	15 (6.8)	0.97 (0.55–1.61)

^g^Sex missing in one patient, *includes rheumatic arthritis

### Quantifying the strength of comorbidity relationships

**Fig. 1:**
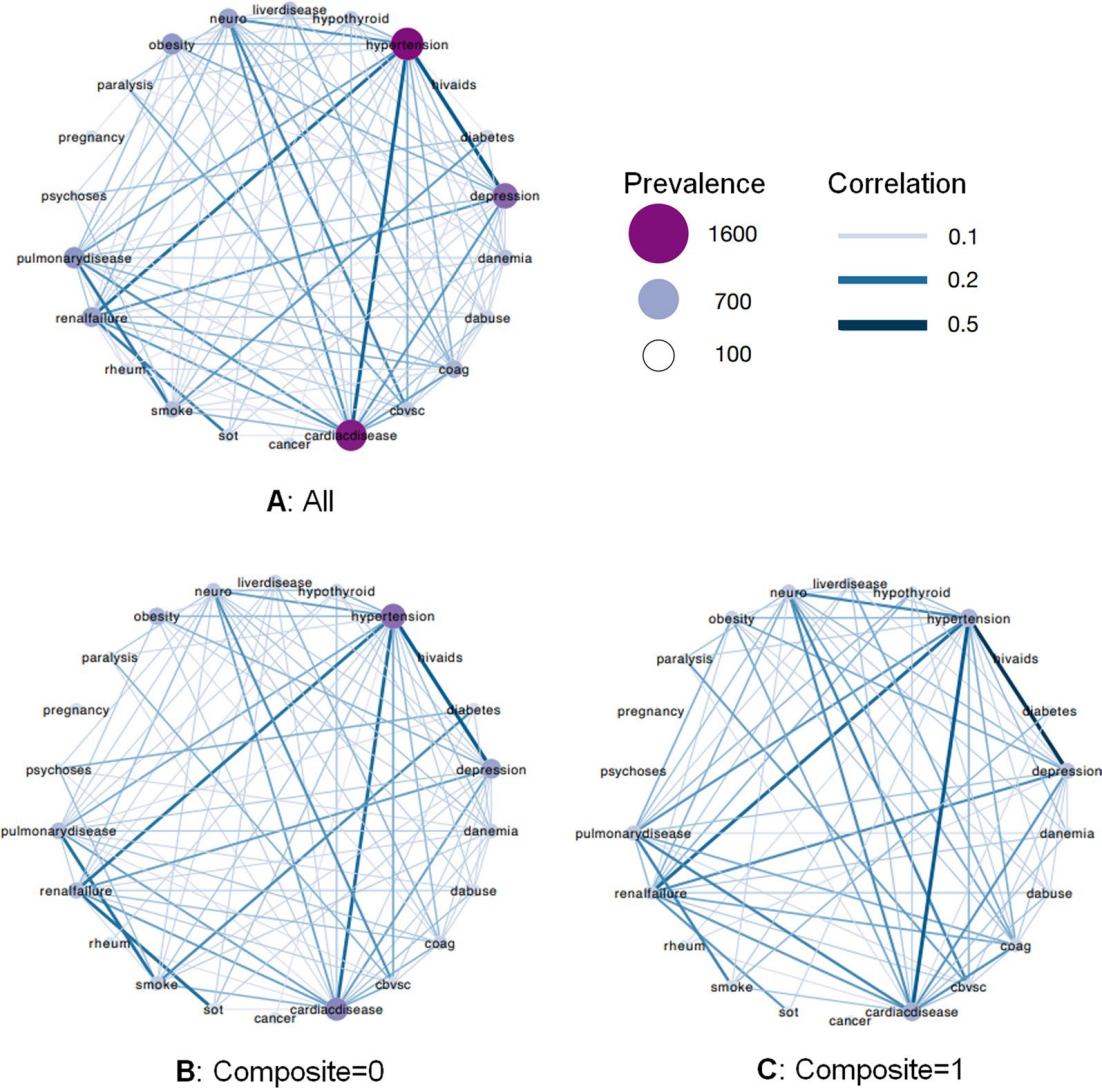
Comparison between the strength of comorbidities observed in patients overall, those who experienced a composite outcome and those that did not. Network includes comorbidities with positive and significant correlation

We quantified the network of comorbidities and visualized correlations that were positive and significant by linking chronic conditions in a visual network (Fig. 1). Figure 1A displays correlations across the total cohort, of which hypertension and heart disease showed the strongest correlation; Fig. 1B displays the correlation network among patients that did not experience a composite outcome; and Fig. 1C describes the correlation network of comorbidities for patients that experienced a composite outcome. Figure 1B and C together suggest that hypertension, diabetes, cardiac disease, renal failure, neurological disease, and depression, among other diseases, tend to be more comorbid in individuals that experienced a composite outcome than in those that did not. The comorbidity network structure in Fig. 1A summarized and reflected well-known associations with COVID-19 hospitalizations, while suggesting that the interactions between comorbidities are expressed differently in those with more severe and deadly cases.

### Quantifying changes in hospitalization, ICU admission and in-hospital mortality

**Fig. 2:**
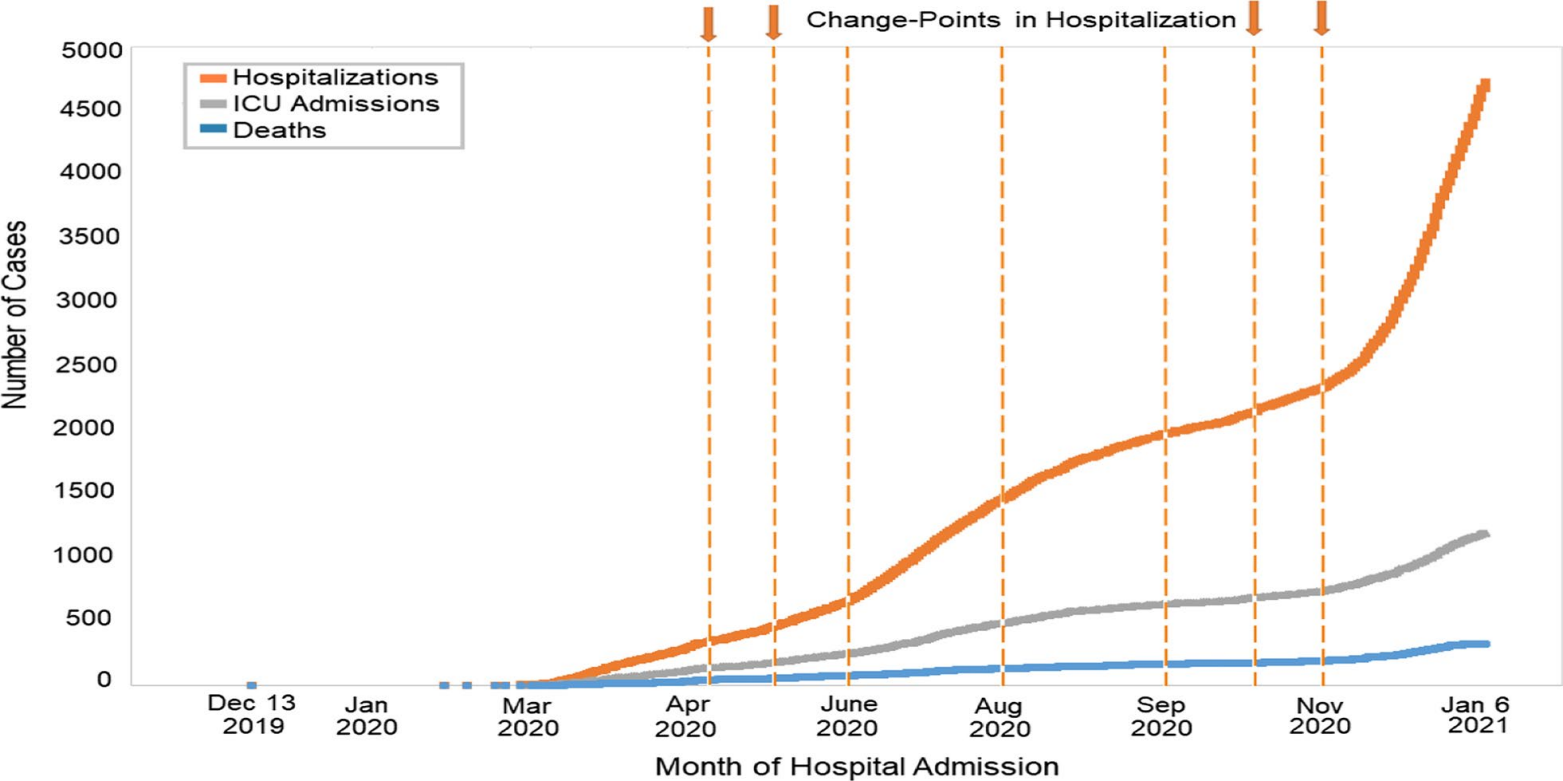
Cumulative Number of Patients with COVID-19 Hospitalized, admitted to the ICU, and Died in UC Health Hospitals, December 13, 2019 to January 6, 2021. Dashed vertical lines correspond to change points in hospitalization that occurred in April 18, May 18, June 25, August 14, September 11, October 15, and November 21, 2020

Figure 2 shows the daily cumulative COVID-19-related hospital admissions, ICU admissions, and deaths during hospitalization. Change-point analysis showed that April 18, May 18, June 25, August 14, September 11, October 15, and November 21 corresponded to significant abrupt changes in the number of hospitalizations. Similar analysis for the number of ICU admissions and deaths are included in Additional file 1: Figure S1. Similar abrupt changes in ICU admissions occurred on the same dates as those for hospitalization; death trends changed on April 15, May 26, July 3, August 6, September 13, October 16, and November 22, 2020.

### Univariable analysis: ICU admission

We identified 1194 (25.2%) patients who were admitted to the intensive care unit with confirmed infection with SARS-CoV-2 (Table 1). In unadjusted univariate analyses, patients of age 55–74 and 75–84 years were more likely to be admitted to the ICU than 18–44 year-olds, and ICU admission was more likely in male patients than female. Hispanic/Latino, Black/African American, and Asian patients were no more or less likely to be admitted to the ICU once in the hospital than White patients, though Other races/ethnicities were (OR 1.36, 95% CI 1.08–1.71). Among the common comorbidities, the most likely to be admitted to the ICU were those patients with neurological conditions (OR 2.84, 95% CI 2.40–3.35), coagulopathy (OR 2.66, 95% CI 2.22–3.19), and cerebrovascular disease (OR 2.49, 95% CI 2.00–3.09). Other high-risk chronic conditions included cardiac disease, pulmonary disease, diabetes, hypertension, obesity, liver disease, and renal failure.

### Univariable analysis: in-hospital deaths

A total of 329 (7.0%) died during hospitalization (Table 1). The risk of in-hospital mortality increased significantly with age, particularly among patients of age 85 years and older (OR 10.25, 95% CI 5.49–21.34) compared to 18–34 year-olds. Male patients were more likely to die in-hospital than female (OR 1.38, 95% CI 1.09–1.74). Hispanic/Latino, and Other patients were no more or less likely to die than White; however, Asians showed an increased likelihood of in-hospital mortality (OR 1.54, 95% CI 1.05–2.24) and Black/African Americans were trending in the same direction (OR 1.44, 95% CI 0.91–2.21), albeit with a smaller sample (n = 46 vs. 31, respectively). The presence of 12 out of the 22 comorbidities investigated were significantly associated with increased odds of in-hospital mortality: neurological conditions (OR 4.31, 95% CI 3.40–5.46), coagulopathy (OR 3.21, 95% CI 2.46–4.14), and cerebrovascular disease (OR 3.19, 95% CI 2.35–4.28), had the most pronounced association with in-hospital mortality. Diabetes, hypertension, liver disease, pulmonary disease, renal failure, and a current smoking history also increased the odds of in-hospital mortality.

### Multivariable analysis: composite outcome

**Fig. 3:**
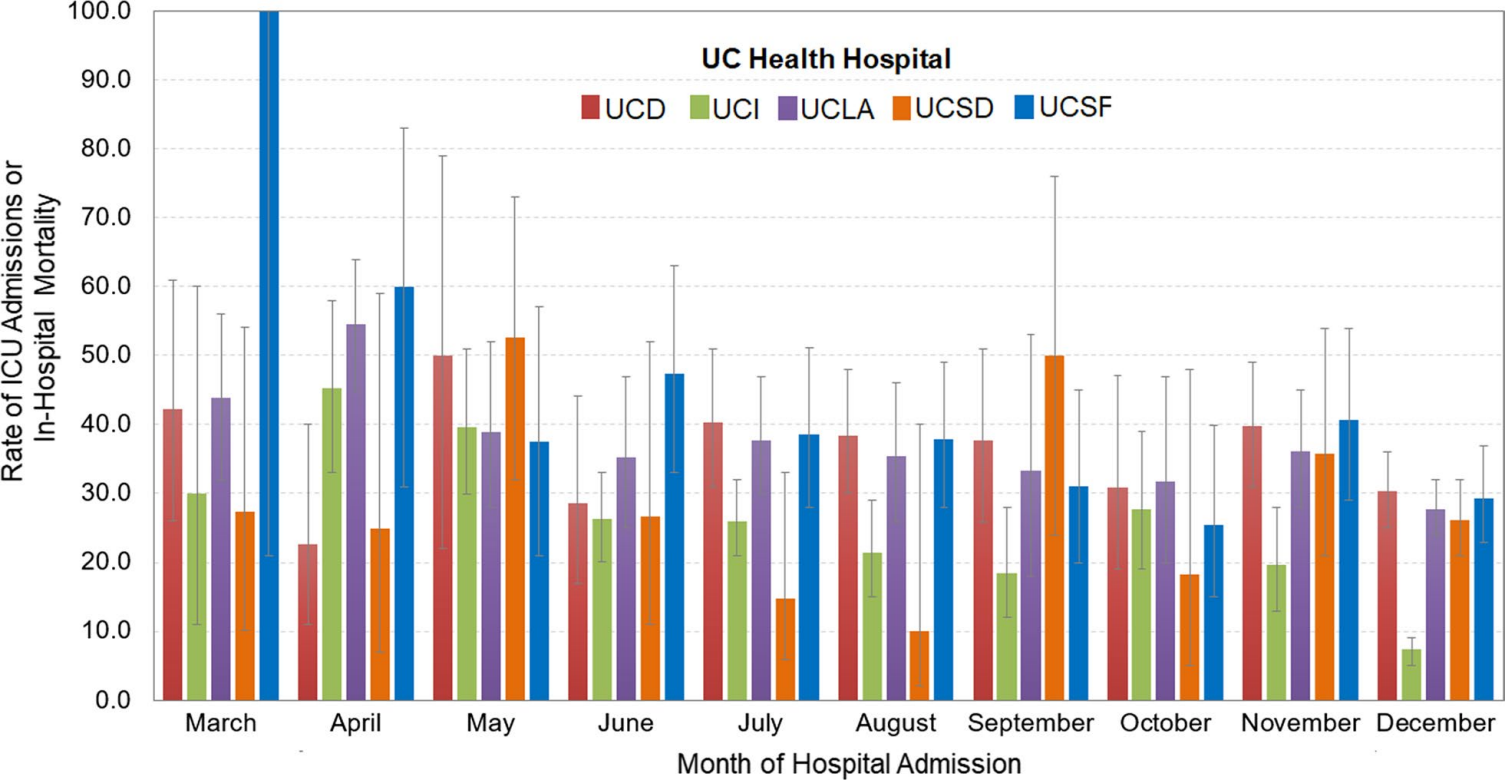
Rate (95% Confidence Intervals) of Patients Who Experienced a Composite Outcome of ICU Admission and/or Death, During Hospitalization for COVID-19 in UC Health Hospitals, 2020

**Fig. 4:**
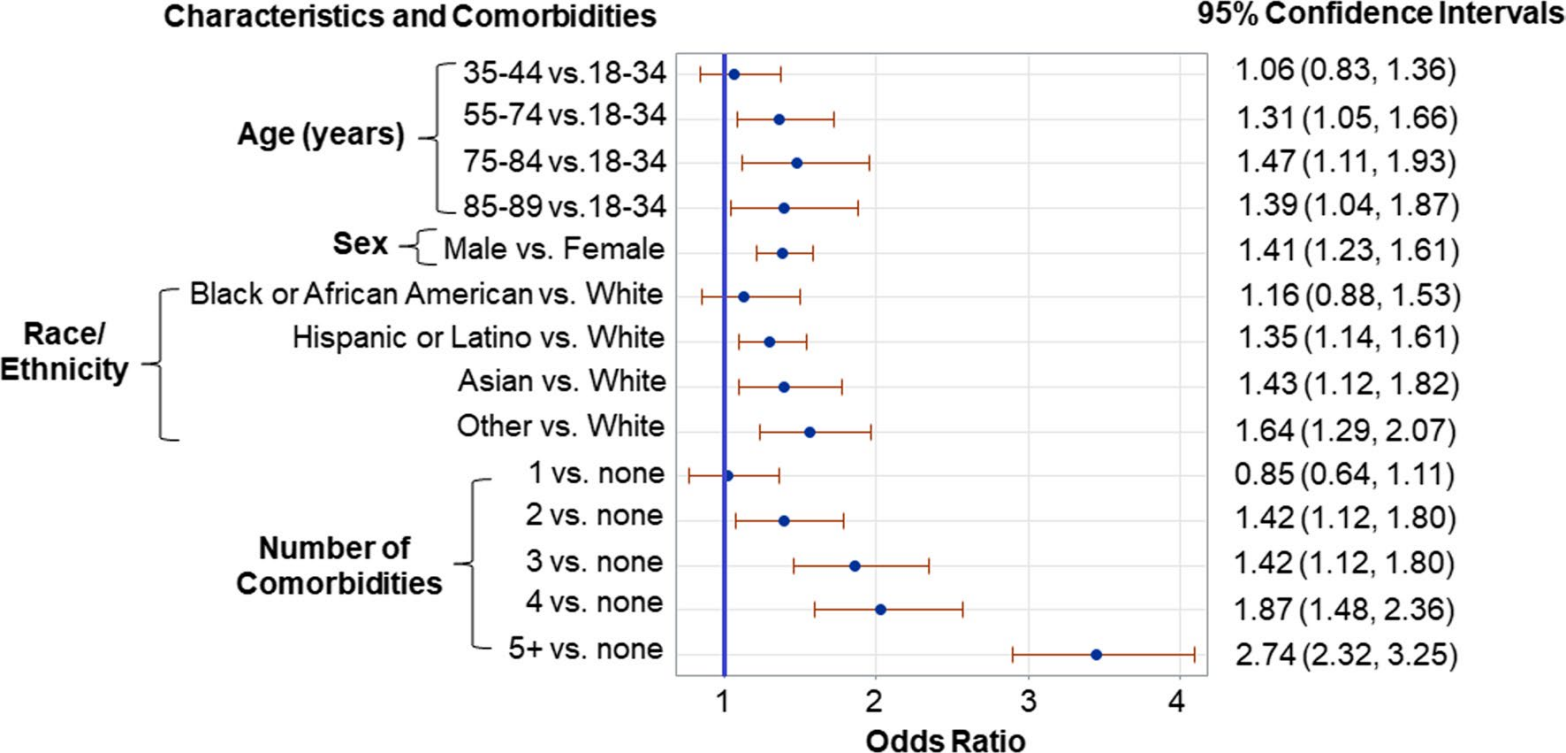
Multivariable Logistic Regression Results for Assessing Factors Associated with the Composite Outcome of In-Hospital Mortality and/or Admission to the ICU Among Patients Hospitalized for COVID-19 in UC Health Hospitals, December 2019 to January 2021

Overall, 27.4% (1298/4730) of patients requiring a hospitalization for COVID-19 were admitted in the ICU and/or died during their hospitalization, defined here as a “composite outcome.” The rate of patients with a composite outcome differed across the five UC Health Hospitals (UCD: 33.9%, 287/846; UCI: 18.2%, 285/1564; UCLA: 33.1%, 424/1283; UCSD: 22.9%, 108/471; UCSF: 34.2%, 194/566, p < 0.001). Composite outcome rates also vary by month of hospital admission for 2020, with an outlier in March of 2020 at UCSF, due to a single hospitalization requiring ICU admission (Fig. 3). In analysis adjusted for age and the number of relevant comorbidities, we found that older patients (85 + vs. 18–34, OR 1.39, 95% CI 1.04–1.87), male (OR 1.41, 95% CI 1.23–1.61), Hispanic/Latino (OR 1.35, 95% CI 1.14–1.61), and Asian (OR 1.43, 95% CI 1.13–1.82) were more likely to be admitted to the ICU and/or die during hospitalization (Fig. 4). Patients with higher number of comorbidities were also more likely to experience a composite outcome (≥ 5 vs. none, OR 1.74, 95% CI 2.32–3.25).

## Discussion

In this study of 4730 patients with COVID-19 requiring hospitalization at five hospitals in California, 25.2% were admitted to the ICU, and 7.0% died. Patients were older (20% greater than 75 years), largely Hispanic/Latino (45.7%), with moderate representation of White (25.8%), Asian (10.3%), and Black/African American (7.2%). The 1-year cumulative ICU admission rate for COVID-19 is equitable to previous reports, but the in-hospital mortality rate is smaller than observed in case studies from earlier in course of the pandemic: ICU admission rates ranged from 14.2% to 35.7% in March–May 2020, where mortality rates ranged from 21.0% to 29.1% over the same period [2, 9, 11, 21]. Our study captured the experience of patients hospitalized for COVID-19 in five hospitals in California, across which the rates of composite outcome ranged from 18.2% to 34.2%. The variability of this outcome by hospital is likely influenced by differences in patient severity and treatment practices for COVID-19 across hospital settings, which have evolved throughout 2020, and regional fluctuations in the scope of the pandemic.

Differences observed in mortality and severity of COVID-19 among patients that require hospitalization are probably multifactorial. Our sample was drawn from a longer time period, which would include effects from improved treatment options and resource availability that were frequently lacking during earlier stages. Additionally, geographic differences in the distribution of races/ethnicities and the prevalence of chronic conditions that appear to increase the risk of severe illness may contribute to differences in hospital outcomes across the country. Criteria for hospital admission may have changed throughout this pandemic and likely will be driven, at least partially, by the availability of resources and ICU beds, which have fluctuated as well. Despite the documented differences in the outcomes of hospitalized COVID-19 patients, trends in these outcomes across the five hospitals during the study period are consistent, with reasonable fluctuations of the outcomes assessed.

The associations with older age, comorbidities, and race/ethnicity with poor outcomes linked to COVID-19 hospitalization are largely consistent with previous findings. This study also highlighted the increased risk of poor outcomes in the Asian population of California. In most states and counties, the relatively small proportion of Asian in the population makes awareness of cases and deaths in this community more challenging, as general samples large enough to make conclusions from are difficult to come by. California is one of few states with a sufficiently sizable Asian population for their COVID-19 burden to become unmistakable in the aggregate. There is growing recognition of the burden of COVID-19 among Asian, but data on outcomes among Asian ethnic subgroups remain extremely limited. A recent systematic review and meta-analysis of 50 studies from the US and United Kingdom found higher risk of COVID-19 infection among Asians and Blacks compared to Whites, and possibly an increased risk of ICU admission and death only among Asians compared to Whites [22]. Further studies are needed to investigate the impact of COVID-19 infection and severity in Asian populations, particularly addressing potential differences in Asian ethnic subgroups at a broader geographic scale. These investigations will be critical to appropriately allocate resources to hardest hit communities, including testing and communication regarding seeking care as well as public health policies to mitigate risk factors and improve health equity.

The composite outcome in this study involved an ICU admission and/or in-hospital mortality, which occurred in 27.5% of patients. We reported rates only for patients who were discharged alive or died during the hospitalization by the end of the study period. 61.8% of patients had 1 or more relevant comorbidities and nearly 23% had 5 or more of these pre-existing conditions, which substantially increased the odds of a poor outcome. Using a network-based approach, we found that hypertension, cardiac disease, diabetes, and renal failure are positively and significantly associated to each other in the overall population, a relationship even more evident in patients with a composite outcome.

This study has several limitations. Our findings represent the experience of five hospitals within the UC Health system and therefore may have limited external generalizability to other health care settings, especially outside of California. Since our sample was drawn from electronic medical records and not full chart reviews, our data was also missing reported symptoms and the patients’ stated reasons for seeking COVID-19 tests, which could be one or a combination of the presence of COVID-19 symptoms, notification of exposure through contact tracing, or seeking a test in lieu of observing a 14-day quarantine period after travel. Since our analysis was based on ICU admission, death, or discharge over the course of a single hospital stay, no losses to follow-up were represented in our data. Such a large cohort followed over an extended time of period will necessarily include aggregation biases due to differential test availability, hospital admission practices, and documentation across the five medical centers, especially as public health advice evolved over the observation period. This could very well have obscured evolving demographic trends as the pandemic developed, or introduced a confounding bias in crude measures. Finally, this study does not address or account for the possibility of patients dying elsewhere. Cases who were tested at UC Health and sought care outside the system were not captured in this database, meaning any subsequent hospitalizations, ICU admissions, or mortality events of positive, confirmed cases outside the UC Health system would not have been captured either. Notwithstanding these limitations, this study provides comparative epidemiologic characteristics of a diverse and underrepresented population of patients admitted to the hospital with COVID-19 over an entire year of the pandemic. Particularly relevant is the captured experience of Asians hospitalized due to COVID-19, and the differences in demographic characteristics and associations of comorbidities with each other and with poor outcomes.

## Conclusions

In this study, we found that older patients, with multiple comorbidities, identifying as Hispanic/Latino or Asian were more likely to be admitted to the intensive care unit and/or die during hospitalization at five UC Health medical centers. The overall mortality rate observed is significantly lower than what has been documented in hospitalized patients with COVID-19 from the early stages of the pandemic, despite the reported high levels of comorbidities. These findings provide additional evidence of the impact of COVID-19 in system of hospitals in California. While the comparatively low mortality rate is reassuring, the differential impact on racial/Ethnic minorities requires the implementation of an equitable solution.

## Supplementary Information

**Additional file 1: Table S1**. Severe acute respiratory syndrome coronavirus 2 Lab Tests. **Table S2**. International classification of disease codes for comoridities. **Appendix S1**. Correlation Network of comorbidities. **Appendix S2**. Change-point detection analysis of hospitalizations, ICU admissions, and In-Hospital deaths. **Figure S1**. Fits of data and change points.

## Data Availability

The data that support the findings of this study are made available by University of California Office of the President and the University of California. Biomedical, Research, Acceleration, Integration, and Development (UC BRAID) but restrictions apply to the availability of these data, which were used under license for the current study, and so are not publicly available. Data are however available from the corresponding author upon reasonable request and with permission of the UC BRAID.

## Abbreviations

SARS-CoV-2: Severe Acute Respiratory Syndrome Coronavirus 2
COVID-19: Coronavirus disease 2019
UC Health: University of California Five Academic Medical Centers
UCD: University of California Davis, Medical Center
UCI: University of California Irvine, Medical Center
UCLA: University of California Los Angeles, Medical Center
UCSD: University of California San Diego, Medical Center
UCSF: University of California San Francisco, Medical Center
ICU: Intensive Care Unit
ICU Days: Days spent in the Intensive Care Unit
LOS: Days spent in the hospital before discharge or death
ICD-10-CM: International Classification of Diseases, 10th Revision, Clinical Modification
AIAN: American Indian/Alaskan Native
